# Trends in healthcare utilization and costs associated with acute otitis media in the United States during 2008–2014

**DOI:** 10.1186/s12913-018-3139-1

**Published:** 2018-05-02

**Authors:** Sabine Tong, Caroline Amand, Alexia Kieffer, Moe H. Kyaw

**Affiliations:** 1 IVIDATA Stats, 79 Rue Baudin, 92300 Levallois-Perret, France; 2 Sanofi, 1 Avenue Pierre Brossolette, 91380 Chilly-Mazarin, France; 3 Sanofi Pasteur, 14 Espace Henry Vallée, 69007 Lyon, France; 4 Sanofi Pasteur, 1 Discovery Drive, Swiftwater, PA 18370 USA

**Keywords:** Healthcare utilization, Healthcare cost, Acute otitis media, United States, Pneumococcal conjugate vaccine, Epidemiology

## Abstract

**Background:**

Acute otitis media (AOM) is the most common cause of pediatric medical visits and antibiotic prescriptions worldwide, but its current impact on the US healthcare system is not clear. The aim of this study was to investigate changes in the incidence of AOM from 2008, just before 13-valent pneumococcal conjugate vaccine was introduced, to 2014 using US insurance records in the Truven MarketScan^®^ database. The study also examined the costs associated with index AOM events during the two most recent years for which data were available (2013–2014).

**Methods:**

AOM cases in the MarketScan database during 2008–2014 were identified using ICD9 diagnosis codes 381.xx and 382.xx. Incidence rates of healthcare utilization related to the index AOM episode were calculated using the annual number of enrolled person-years as the denominator and the number of individuals with AOM as the numerator. AOM-associated costs were calculated as the mean payment per episode during the 2 years from 2013 to 2014.

**Results:**

The overall annual rate of AOM-related healthcare utilization was 60.5 per 1000 person-years and changed little from 2008 to 2014 (range, 58.4–62.6). Most of this was due to office/outpatient visits (55.7 [range, 52.0–58.8] per 1000 person-years). Emergency department/urgent care visits (4.7 [range 3.7–6.3] per 1000 person-years) and hospitalization (0.0 [range, 0.0–0.1] per 1000 person-years) contributed little. The rate of AOM-related healthcare utilization per 1000 person-years was highest in the youngest children and declined with age (474.3 for < 1 year, 503.9 for 1 year, 316.3 for 2–4 years, 94.9 for 5–17 years, 33.1 for 18–49 years, 28.6 for 50–64 years, 23.7 for 65–74 years, 20.2 for 75–84 years, and 16.1 for ≥85 years). The mean cost per AOM episode in 2013–2014 (95% confidence interval) was $199.0 (198.4–199.6) for office or outpatient visits, $329.6 (328.2–331.0) for emergency department/urgent care visits, and $1592.9 (1422.0–1763.8) for hospitalization.

**Conclusions:**

In the US, AOM-associated healthcare utilization and costs remain substantial. More effective preventive measures such as new vaccines are needed to reduce the burden of AOM.

## Background

Every year, an estimated 709 million episodes of acute otitis media (AOM) occur globally, half of which are in children < 5 years of age [1]. By 3 years of age, at least 80% of children are expected to have had at least one episode [2]. This makes AOM the most common pediatric infectious disease and most common reason for medical visits, antibiotic prescriptions, and hearing impairment in children [2].

*Streptococcus pneumoniae*, non-typable *Haemophilus influenzae*, and *Moraxella catarrhalis* are the most common causes of AOM [3]. Over the last several decades, a variety of vaccines have been introduced in the US to *control S. pneumoniae*, including a 14-valuent pneumococcal polysaccharide vaccine in 1977, the current 23-valuent pneumococcal polysaccharide vaccine in 1983, a 7-valent pneumococcal conjugate vaccine (PCV7) in 2000, and a 13-valent pneumococcal conjugate vaccine (PCV13) in 2010 [4]. Vaccines against non-typeable *H. influenza*e and *M. catarrhalis*, however, are not currently available in the US. PCV7 greatly reduced the incidence of invasive pneumococcal disease and *S. pneumoniae* carriage [5], but the extent to which it affected AOM rates remains unclear [6], and even after PCV13 was introduced in 2010, *S. pneumoniae* remained one of the most frequent causes of AOM, accounting for 26–36% of cases in the US [7].

Further data on the real burden of AOM and its impact on the healthcare system are needed to assess the impact of recent interventions, especially PCV13, on AOM and to inform the development and use of new vaccines and other preventive measures. The aim of the current study was therefore to investigate changes in the incidence of AOM from 2008, just before PCV13 was introduced, to 2014, the most recent year for which data were available, based on US insurance records in the Truven MarketScan database. The study also examined the costs associated with index AOM events in the US during the two most recent years for which data were available (2013–2014).

## Methods

### Study design

This was a retrospective analysis of AOM-related healthcare utilization in the US using data extracted from the Truven Health MarketScan^®^ Commercial Claims and Encounters database [8]. The co-primary objectives were (a) to determine the annual and monthly incidence rates of AOM-related healthcare utilization during 2008–2014 and (b) to determine the AOM-associated costs during the two most recent years (2013–2014) due to hospitalization, emergency department (ED)/urgent care (UC) visits, and outpatient visits.

### Data source and extraction

The MarketScan database contains information on individuals in the US who are insured commercially (i.e. privately) or through the Medicare program [8]. The database collects data on paid claims from employers, health plans, and state-level Medicaid agencies using a nationwide convenience sample. It contains complete longitudinal records of patient demographics, inpatient services, outpatient services, long-term care, and prescription drug claims. The database includes an average of 48,982,662 individuals per year and covers all census regions of the US. Accordingly, the database is considered representative of the US population with employer-provided health insurance [9, 10] and is widely used to understand the burden and healthcare utilization for different illnesses in the US. All database records are de-identified and fully compliant with US patient confidentiality requirements, including the Health Insurance Portability and Accountability Act of 1996. Accordingly, ethical approval was not required for this study.

Data from January 1, 2008 to December 31, 2014 were extracted. Only data from individuals in the enrollment tables were included. AOM episodes were identified as (a) inpatient admissions with the principal diagnosis of AOM (International Classification of Diseases, 9th revision, Clinical Modification [ICD-9-CM] code 381.xx, or 382.xx) or (b) outpatient visits with a primary or secondary diagnosis of AOM (ICD-9-CM 381.xx, or 382.xx). In addition, the first consultation had to be more than 28 days after any previous consultation with the same diagnosis code. The index episode was defined as the first episode of AOM occurring during the calendar year. Data extracted included total enrollment numbers for each year; demographic data, including age, sex, geographic region (Northeast, North Central, South, West, or unknown), and insurance type (commercial, Medicare); and amounts paid by insurers, health plans, and patients for adjudicated claims.

### Outcome measures and definitions

Outcome measures included the number of AOM cases (overall and by insurance type and geographical region); demographics for the index visit (mean and median age, age range, and sex distribution); annual incidence of AOM-related healthcare utilization overall and by setting (hospitalization, ED/UC visits, and outpatient visits) of the index visit for all ages and each age group; proportions of AOM cases for each setting type for all ages and each age group; and monthly incidence rates of AOM. For patients transferred to several services on the same day, the setting was defined as the most severe (i.e. hospitalization > ED/UC > outpatient). AOM-associated costs for the 2 years from 2013 to 2014 were determined overall and by setting for the index visit and all follow-up visits with the same diagnosis code occurring within 28 days. Costs were based on paid amounts of adjudicated claims, including insurer and health plan payments and patient cost-sharing in the form of copayments, deductibles, and coinsurance. Total costs were estimated as the sum of the costs for the individual settings.

### Statistical analysis

Annual incidence rates of AOM were per 1000 person-years, calculated as 1000 × [annual number of patients with an AOM episode] ÷ [annual number of total enrolled person-years in the MarketScan databases]. Monthly incidence rates for each year were calculated per 1000 person-months. Proportions were calculated as 100% × [number of index visits for each setting] ÷ [number of total index visits]. Costs related to the index episode of AOM were calculated as the mean payment per episode during the two years from 2013 to 2014. The 95% confidence intervals (CIs) for incidence rates and costs were calculated using a normal approximation. All analyses were performed using SAS^®^ Enterprise Guide 7.1 (SAS Institute, Cary, NC, USA).

## Results

### AOM cases

Between January 1, 2008 and December 31, 2014, the database included an average of 41,610,536 person-years each year. On average, 2,520,207 cases of AOM were diagnosed each year.

### Demographics of AOM cases

Slightly less than half (range, 45.4%–46.8%) of AOM patients were male, and the mean age was 20.1 years (Table 1). Overall, children and adolescents (< 18 years) represented 61% of AOM cases, non-elderly adults (18–64 years) 36%, and elderly adults (≥ 65 years) only 3%. For all years, more than 96% of patients with AOM were commercially insured. Most cases were reported in the South (41.4% overall) and North Central region (24.8% overall), although from 2008 to 2014, the proportion increased for the Northeast region (from 9.1% to 20.5%) and decreased (from 50.8% to 38.8%) for the South region.

**Table 1:** Demographic characteristics of AOM patients

Variable	2008	2009	2010	2011	2012	2013	2014	Mean (range)
AOM patients, n	1,862,125	2,332,888	2,519,193	3,014,969	2,996,079	2,403,475	2,512,720	2,520,207 (1,862,125–3,014,969)
Age (y)								
Mean (SD)	18.7 (20.7)	19.1 (20.9)	19.7 (21.3)	19.9 (21.3)	20.2 (21.3])	21.3 (22.0)	22.0 (21.9)	20.1 (18.7–22.0)
Median	8	9	9	9	10	11	12	10 (8–12)
Age range, n (%)								
< 1 y	166,785 (9.0)	193,895 (8.3)	203,662 (8.1)	225,800 (7.5)	225,670 (7.5)	172,411 (7.2)	175,355 (7.0)	7.8 (7.0–9.0)
1 y	174,212 (9.4)	209,180 (9.0)	223,144 (8.9)	254,902 (8.5)	250,197 (8.4)	195,332 (8.1)	198,919 (7.9)	8.6 (7.9–9.4)
2–4 y	342,888 (18.4)	420,827 (18.0)	450,393 (17.9)	533,726 (17.7)	516,963 (17.3)	401,101 (16.7)	397,208 (15.8)	17.4 (15.8–18.4)
5–17 y	508,926 (27.3)	652,434 (28.0)	692,930 (27.5)	848,642 (28.1)	824,756 (27.5)	639,879 (26.6)	649,593 (25.9)	27.3 (25.9–28.1)
18–49 y	436,012 (23.4)	556,016 (23.8)	603,437 (24.0)	730,878 (24.2)	753,916 (25.2)	613,892 (25.5)	682,419 (27.2)	24.8 (23.4–27.2)
50–64 y	192,979 (10.4)	243,496 (10.4)	275,264 (10.9)	330,137 (10.9)	339,436 (11.3)	291,402 (12.1)	326,341 (13.0)	11.3 (10.4–13.0)
65–74 y	22,369 (1.2)	33,277 (1.4)	41,710 (1.7)	54,066 (1.8)	51,672 (1.7)	56,053 (2.3)	52,674 (2.1)	1.7 (1.2–2.3)
75–84 y	13,951 (0.7)	18,220 (0.8)	21,829 (0.9)	27,581 (0.9)	24,824 (0.8)	24,976 (1.0)	22,466 (0.9)	0.9 (0.7–1.0)
≥ 85 y	4003 (0.2)	5543 (0.2)	6824 (0.3)	9237 (0.3)	8645 (0.3)	8429 (0.4)	7745 (0.3)	0.3 (0.2–0.4)
Male, n (%)	871,434 (46.8)	1,090,070 (46.7)	1,175,316 (46.7)	1,406,050 (46.6)	1,392,011 (46.5)	1,104,083 (45.9)	1,141,612 (45.4)	46.4 (45.4–46.8)
Insurance, n (%)								
Commercial ^a^	1,820,948 (97.8)	2,274,874 (97.5)	2,447,618 (97.2)	2,922,112 (96.9)	2,909,203 (97.1)	2,312,057 (96.2)	2,428,161 (96.6)	97.0 (96.2–97.8)
Medicare ^b^	41,177 (2.2)	58,014 (2.5)	71,575 (2.8)	92,857 (3.1)	86,876 (2.9)	91,418 (3.8)	84,559 (3.4)	3.0 (2.2–3.8)
US geographic region, n (%)								
Northeast	169,659 (9.1)	285,197 (12.2)	368,765 (14.6)	565,050 (18.7)	540,654 (18.0)	438,273 (18.2)	515,136 (20.5)	15.9 (9.1–20.5)
North Central	481,564 (25.9)	632,555 (27.1)	648,970 (25.8)	784,658 (26.0)	746,438 (24.9)	539,270 (22.4)	546,186 (21.7)	24.8 (21.7–27.1)
South	945,338 (50.8)	1,105,089 (47.4)	1,054,087 (41.8)	1,099,741 (36.5)	1,144,577 (38.2)	880,547 (36.6)	974,374 (38.8)	41.4 (36.5–50.8)
West	256,447 (13.8)	306,441 (13.1)	416,690 (16.5)	471,455 (15.6)	493,978 (16.5)	473,180 (19.7)	408,917 (16.3)	15.9 (13.1–19.7)
Unknown	9117 (0.5)	3606 (0.2)	30,681 (1.2)	94,065 (3.1)	70,432 (2.4)	72,205 (3.0)	68,107 (2.7)	1.9 (0.2–3.1)

*Abbreviations*: *SD* standard deviation

^a^Includes active employees and their dependents, early (non-Medicare) retirees and their dependents, and individuals covered under the Consolidated Omnibus Budget Reconciliation Act

^b^Includes retirees (> 65 y), their dependents, and younger people with disabilities

### Incidence of AOM

The mean annual incidence rate of AOM was 60.5 per 1000 person-years (Table 2). Based on this and the US population for 2016 (322,762,018), an estimated 19.5 million patients suffer from AOM each year.

**Table 2:** Incidence of index AOM visits by setting, age group, and year

Rate of AOM cases per 1000 person-years (95% CI)									
Age group	Setting	2008	2009	2010	2011	2012	2013	2014	2008–2014 Mean (range)
Overall	All	59.1 (59.0–59.2)	62.6 (62.5–62.7)	60.0 (60.0–60.1)	62.3 (62.2–62.4)	61.8 (61.7–61.8)	59.2 (59.1–59.2)	58.4 (58.3–58.5)	60.5 (58.4–62.6)
	Hospitalized	0.0 (0.0–0.0)	0.0 (0.0–0.0)	0.1 (0.1–0.1)	0.0 (0.0–0.0)	0.0 (0.0–0.0)	0.0 (0.0–0.0)	0.0 (0.0–0.0)	0.0 (0.0–0.1)
	ED/UC	3.7 (3.6–3.7)	3.7 (3.7–3.8)	3.9 (3.9–3.9)	4.5 (4.5–4.5)	5.1 (5.1–5.1)	5.7 (5.6–5.7)	6.3 (6.3–6.4)	4.7 (3.7–6.3)
	Outpatient	55.4 (55.3–55.5)	58.8 (58.7–58.9)	56.0 (56.0–56.1)	57.7 (57.7–57.8)	56.6 (56.6–56.7)	53.5 (53.4–53.5)	52.0 (52.0–52.1)	55.7 (52.0–58.8)
< 1 y	All	499.4 (497.0–501.8)	502.3 (500.0–504.5)	486.7 (484.6–488.8)	475.4 (473.4–477.3)	476.5 (474.5–478.5)	448.3 (446.2–450.4)	431.8 (429.8–433.9)	474.3 (431.8–502.3)
	Hospitalized	0.4 (0.4–0.5)	0.4 (0.4–0.5)	1.2 (1.1–1.3)	0.5 (0.4–0.5)	0.5 (0.4–0.5)	0.4 (0.3–0.4)	0.4 (0.3–0.5)	0.5 (0.4–1.2)
	ED/UC	29.9 (29.3–30.5)	29.9 (29.4–30.5)	29.0 (28.5–29.5)	31.3 (30.8–31.8)	32.3 (31.8–32.9)	32.4 (31.8–32.9)	30.8 (30.3–31.4)	30.8 (29.0–32.4)
	Outpatient	469.0 (466.7–471.3)	471.9 (469.8–474.1)	456.5 (454.5–458.6)	443.7 (441.8–445.6)	443.7 (441.8–445.6)	415.6 (413.6–417.6)	400.6 (398.7–402.6)	443.0 (400.6–471.9)
1 y	All	495.8 (493.4–498.1)	515.5 (513.3–517.7)	506.5 (504.4–508.6)	520.5 (518.5–522.5)	513.3 (511.3–515.3)	496.8 (494.6–499.0)	478.6 (476.5–480.7)	503.9 (478.6–520.5)
	Hospitalized	0.3 (0.2–0.3)	0.3 (0.3–0.4)	1.2 (1.1–1.3)	0.4 (0.3–0.4)	0.3 (0.2–0.3)	0.3 (0.3–0.4)	0.3 (0.3–0.4)	0.4 (0.3–1.2)
	ED/UC	34.9 (34.2–35.5)	35.7 (35.1–36.3)	35.6 (35.0–36.2)	39.6 (39.1–40.2)	40.3 (39.7–40.8)	42.5 (41.8–43.1)	43.1 (42.5–43.8)	38.8 (34.9–43.1)
	Outpatient	460.6 (458.4–462.9)	479.5 (477.4–481.6)	469.8 (467.7–471.8)	480.5 (478.6–482.5)	472.8 (470.8–474.7)	454.0 (451.9–456.1)	435.1 (433.1–437.1)	464.6 (435.1–480.5)
2–4 y	All	309.2 (308.1–310.2)	325.1 (324.1–326.1)	313.0 (312.1–313.9)	328.3 (327.5–329.2)	323.7 (322.8–324.6)	314.9 (314.0–315.9)	299.8 (298.8–300.7)	316.3 (299.8–328.3)
	Hospitalized	0.1 (0.1–0.1)	0.1 (0.1–0.1)	0.5 (0.5–0.6)	0.2 (0.1–0.2)	0.2 (0.1–0.2)	0.2 (0.1–0.2)	0.2 (0.2–0.2)	0.2 (0.1–0.5)
	ED/UC	19.0 (18.8–19.3)	19.7 (19.5–20.0)	19.8 (19.6–20.0)	23.1 (22.8–23.3)	24.6 (24.4–24.8)	27.0 (26.7–27.3)	27.4 (27.1–27.7)	22.9 (19.0–27.4)
	Outpatient	290.0 (289.0–291.0)	305.2 (304.3–306.2)	292.7 (291.8–293.6)	305.1 (304.3–306.0)	298.9 (298.1–299.8)	287.7 (286.8–288.7)	272.2 (271.3–273.1)	293.1 (272.2–305.2)
5–17 y	All	89.1 (88.8–89.3)	98.4 (98.2–98.6)	93.1 (92.8–93.3)	100.9 (100.7–101.1)	98.4 (98.2–98.6)	93.7 (93.5–94.0)	90.5 (90.3–90.7)	94.9 (89.1–100.9)
	Hospitalized	0.0 (0.0–0.0)	0.0 (0.0–0.0)	0.1 (0.1–0.1)	0.0 (0.0–0.0)	0.0 (0.0–0.0)	0.0 (0.0–0.0)	0.0 (0.0–0.0)	0.0 (0.0–0.1)
	ED/UC	5.3 (5.2–5.4)	5.8 (5.7–5.9)	6.0 (6.0–6.1)	7.3 (7.2–7.3)	8.0 (7.9–8.0)	8.9 (8.8–9.0)	9.7 (9.6–9.8)	7.3 (5.3–9.7)
	Outpatient	83.7 (83.5–84.0)	92.6 (92.3–92.8)	86.9 (86.7–87.1)	93.6 (93.4–93.8)	90.4 (90.2–90.6)	84.8 (84.6–85.1)	80.8 (80.6–81.0)	87.5 (80.8–93.6)
18–49 y	All	30.4 (30.3–30.5)	33.2 (33.1–33.3)	32.1 (32.0–32.2)	33.7 (33.7–33.8)	33.9 (33.8–34.0)	33.5 (33.4–33.6)	34.5 (34.5–34.6)	33.1 (30.4–34.5)
	Hospitalized	0.0 (0.0–0.0)	0.0 (0.0–0.0)	0.0 (0.0–0.1)	0.0 (0.0–0.0)	0.0 (0.0–0.0)	0.0 (0.0–0.0)	0.0 (0.0–0.0)	0.0 (0.0–0.0)
	ED/UC	2.3 (2.2–2.3)	2.3 (2.3–2.3)	2.6 (2.5–2.6)	3.0 (3.0–3.0)	3.7 (3.6–3.7)	4.4 (4.3–4.4)	5.2 (5.2–5.2)	3.3 (2.3–5.2)
	Outpatient	28.1 (28.0–28.2)	30.9 (30.8–31.0)	29.5 (29.4–29.6)	30.7 (30.6–30.8)	30.2 (30.1–30.3)	29.1 (29.0–29.2)	29.3 (29.3–29.4)	29.7 (28.1–30.9)
50–64 y	All	26.0 (25.9–26.1)	27.4 (27.3–27.5)	27.5 (27.4–27.6)	28.7 (28.6–28.8)	29.3 (29.2–29.4)	30.0 (29.9–30.1)	30.9 (30.8–31.0)	28.6 (26.0–30.9)
	Hospitalized	0.0 (0.0–0.0)	0.0 (0.0–0.0)	0.0 (0.0–0.0)	0.0 (0.0–0.0)	0.0 (0.0–0.0)	0.0 (0.0–0.0)	0.0 (0.0–0.0)	0.0 (0.0–0.0)
	ED/UC	1.0 (1.0–1.1)	1.0 (1.0–1.1)	1.2 (1.2–1.3)	1.5 (1.4–1.5)	1.9 (1.8–1.9)	2.3 (2.2–2.3)	2.8 (2.8–2.8)	1.7 (1.0–2.8)
	Outpatient	25.0 (24.9–25.1)	26.4 (26.3–26.5)	26.2 (26.1–26.3)	27.2 (27.1–27.3)	27.4 (27.3–27.5)	27.7 (27.6–27.8)	28.1 (28.0–28.2)	26.9 (25.0–28.1)
65–74 y	All	19.5 (19.3–19.8)	21.6 (21.4–21.9)	22.7 (22.5–22.9)	23.7 (23.5–23.9)	25.0 (24.8–25.2)	26.5 (26.3–26.7)	26.9 (26.6–27.1)	23.7 (19.5–26.9)
	Hospitalized	0.1 (0.1–0.1)	0.1 (0.1–0.1)	0.1 (0.1–0.1)	0.1 (0.1–0.1)	0.1 (0.1–0.1)	0.1 (0.1–0.1)	0.1 (0.1–0.1)	0.1 (0.1–0.1)
	ED/UC	0.6 (0.6–0.7)	0.6 (0.6–0.7)	0.8 (0.8–0.8)	0.9 (0.9–1.0)	1.0 (0.9–1.0)	1.3 (1.3–1.4)	1.6 (1.5–1.7)	1.0 (0.6–1.6)
	Outpatient	18.8 (18.5–19.0)	20.9 (20.7–21.1)	21.8 (21.6–22.0)	22.7 (22.5–22.9)	23.9 (23.7–24.2)	25.1 (24.9–25.3)	25.2 (25.0–25.4)	22.6 (18.8–25.2)
75–84 y	All	17.2 (16.9–17.4)	18.3 (18.0–18.6)	19.3 (19.1–19.6)	20.1 (19.9–20.4)	21.3 (21.0–21.5)	22.5 (22.2–22.7)	22.9 (22.6–23.2)	20.2 (17.2–22.9)
	Hospitalized	0.1 (0.1–0.1)	0.1 (0.1–0.1)	0.1 (0.1–0.1)	0.1 (0.1–0.1)	0.1 (0.1–0.1)	0.1 (0.1–0.1)	0.1 (0.1–0.1)	0.1 (0.1–0.1)
	ED/UC	0.5 (0.5–0.6)	0.5 (0.4–0.5)	0.6 (0.5–0.6)	0.7 (0.7–0.8)	0.7 (0.7–0.8)	0.9 (0.9–1.0)	1.1 (1.0–1.1)	0.7 (0.5–1.1)
	Outpatient	16.5 (16.3–16.8)	17.7 (17.4–18.0)	18.6 (18.4–18.9)	19.3 (19.1–19.6)	20.4 (20.2–20.7)	21.4 (21.1–21.7)	21.7 (21.4–22.0)	19.4 (16.5–21.7)
≥ 85 y	All	13.9 (13.5–14.4)	14.1 (13.8–14.5)	15.6 (15.3–16.0)	16.2 (15.9–16.5)	17.1 (16.7–17.5)	17.6 (17.3–18.0)	17.9 (17.5–18.3)	16.1 (13.9–17.9)
	Hospitalized	0.2 (0.1–0.2)	0.1 (0.1–0.2)	0.2 (0.1–0.2)	0.1 (0.1–0.1)	0.1 (0.1–0.1)	0.1 (0.1–0.1)	0.1 (0.1–0.2)	0.1 (0.1–0.2)
	ED/UC	0.4 (0.3–0.5)	0.4 (0.3–0.4)	0.5 (0.4–0.6)	0.5 (0.4–0.6)	0.6 (0.5–0.6)	0.7 (0.6–0.7)	0.7 (0.6–0.8)	0.5 (0.4–0.7)
	Outpatient	13.4 (12.9–13.8)	13.6 (13.2–14.0)	15.0 (14.6–15.3)	15.6 (15.3–15.9)	16.4 (16.1–16.8)	16.9 (16.5–17.2)	17.1 (16.7–17.5)	15.4 (13.4–17.1)

*Abbreviations*: *ED* emergency department; UC, urgent care

The mean rate was highest in young children and declined with increasing age (474.3 for < 1 year, 503.9 for 1 year, 316.3 for 2–4 years, 94.9 for 5–17 years, 33.1 for 18–49 years, 28.6 for 50–64 years, 23.7 for 65–74 years, 20.2 for 75–84 years, and 16.1 for ≥85 years). For children and adolescents overall (< 18 years of age), the mean rate was 162.3 per 1000 person-years.

**Fig. 1:**
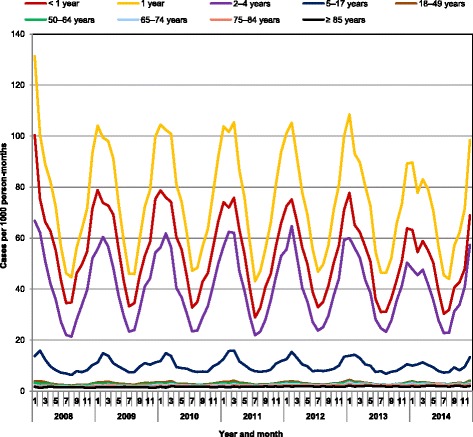
Seasonality of AOM cases in the US, 2008–2014

From 2008 to 2014, overall incidence rates changed little, with no obvious trend (range, 58.4 to 62.6 per 1000 person-years). When assessed by age group, rates decreased over time for the youngest children (≤ 1 year) of age (− 13.5% from 2008 to 2014) and increased for all adult groups (+ 13.5% for 18–49 years, + 18.8% for 50–64 years, + 37.9% for 65–74 years, + 33.1% for 75–84 years, and + 28.8% for ≥85 years from 2008 to 2014). Seasonality of AOM was consistent during these years, with a peak in the winter months in all age groups (Fig. 1).

### Proportion of AOM cases by setting

Overall, most of the index AOM episodes (92.1%) were related to an outpatient visit (Table 3). Only 7.8% of index cases were related to ED or UC visits and only 0.1% were related to hospitalization. This was also the case for each age group, although between 2008 and 2014, the proportion of AOM diagnoses in the outpatient setting decreased slightly (from 93.8% to 89.1% overall) while the proportion made in the ED/UC setting increased from 6.2% to 10.9%.

**Table 3:** Proportion of AOM cases by setting, age group, and year

Proportion of AOM cases (%)									
Age group	Setting	2008	2009	2010	2011	2012	2013	2014	2008–2014 Average (range)
Overall	Hospitalization	0.1	0.1	0.2	0.1	0.1	0.1	0.1	0.1 (0.1–0.2)
	ED/UC visits	6.2	6.0	6.5	7.2	8.2	9.6	10.9	7.8 (6.0–10.9)
	Outpatient visits	93.8	94.0	93.3	92.7	91.7	90.4	89.1	92.1 (89.1–94.0)
< 1 y	Hospitalization	0.1	0.1	0.2	0.1	0.1	0.1	0.1	0.1 (0.1–0.2)
	ED/UC visits	6.0	6.0	6.0	6.6	6.8	7.2	7.1	6.5 (6.0–7.2)
	Outpatient visits	93.9	94.0	93.8	93.3	93.1	92.7	92.8	93.4 (92.7–94.0)
1 y	Hospitalization	0.1	0.1	0.2	0.1	0.1	0.1	0.1	0.1 (0.1–0.2)
	ED/UC visits	7.0	6.9	7.0	7.6	7.8	8.5	9.0	7.7 (6.9–9.0)
	Outpatient visits	92.9	93.0	92.7	92.3	92.1	91.4	90.9	92.2 (90.9–93.0)
2–4 y	Hospitalization	0.0	0.0	0.2	0.0	0.0	0.1	0.1	0.1 (0.0–0.2)
	ED/UC visits	6.2	6.1	6.3	7.0	7.6	8.6	9.1	7.3 (6.1–9.1)
	Outpatient visits	93.8	93.9	93.5	92.9	92.4	91.4	90.8	92.7 (90.8–93.9)
5–17 y	Hospitalization	0.0	0.0	0.1	0.0	0.0	0.0	0.0	0.1 (0.0–0.1)
	ED/UC visits	5.9	5.9	6.5	7.2	8.1	9.5	10.7	7.7 (5.9–10.7)
	Outpatient visits	94.0	94.1	93.4	92.8	91.9	90.5	89.3	92.3 (89.3–94.1)
18–49 y	Hospitalization	0.0	0.0	0.2	0.0	0.0	0.0	0.0	0.1 (0.0–0.2)
	ED/UC visits	7.4	7.0	8.0	9.0	10.8	13.0	15.0	10.0 (7.0–15.0)
	Outpatient visits	92.5	93.0	91.8	91.0	89.2	86.9	84.9	89.9 (84.9–93.0)
50–64 y	Hospitalization	0.1	0.1	0.1	0.1	0.1	0.1	0.1	0.1 (0.1–0.1)
	ED/UC visits	4.0	3.8	4.5	5.1	6.3	7.5	9.1	5.8 (3.8–9.1)
	Outpatient visits	96.0	96.1	95.4	94.9	93.6	92.4	90.9	94.2 (90.9–96.1)
65–74 y	Hospitalization	0.4	0.3	0.4	0.3	0.3	0.3	0.3	0.3 (0.3–0.4)
	ED/UC visits	3.3	3.0	3.5	4.0	3.9	5.1	6.0	4.1 (3.0–6.0)
	Outpatient visits	96.3	96.7	96.1	95.7	95.8	94.7	93.8	95.6 (93.8–96.7)
75–84 y	Hospitalization	0.6	0.6	0.7	0.4	0.5	0.5	0.4	0.5 (0.4–0.7)
	ED/UC visits	2.9	2.7	3.0	3.6	3.4	4.1	4.7	3.5 (2.7–4.7)
	Outpatient visits	96.4	96.8	96.4	96.0	96.1	95.4	94.9	96.0 (94.9–96.8)
≥ 85 y	Hospitalization	1.1	1.0	1.0	0.6	0.6	0.6	0.8	0.8 (0.6–1.1)
	ED/UC visits	2.9	2.7	3.2	3.1	3.3	3.7	3.9	3.3 (2.7–3.9)
	Outpatient visits	96.0	96.3	95.8	96.3	96.1	95.6	95.4	95.9 (95.4–96.3)

*Abbreviations*: *ED* emergency department; UC, urgent care

### Costs of index AOM episodes during 2013–2014

During the 2 years from 2013 to 2014, the total mean cost per AOM episode was $218.3 (95% CI, 217.7–218.9) (Table 4). Outpatient visits accounted for 91.2% of all AOM episodes and an average cost of $199.0 (95% CI, 198.4–199.6) per episode. ED/UC visits accounted for 10.7% of all AOM episodes and an average cost of $329.6 (95% CI, 328.2–331.0) per episode. Hospitalizations accounted for 0.1% of all AOM episodes and an average cost of $1592.9 (95% CI, 1422.0–1763.8) per episode. The cost of AOM ranged from $186.1 to $344.2 for children and adolescents, $177.6 to $188.8 for non-elderly adults, and $201.4 to $218.9 for elderly adults. Based on our estimate of 19.5 million AOM cases each year for the full US population, estimated annual costs would be US$ 4.3 billion.

**Table 4:** Mean costs related to the index AOM episode by setting and age group during 2013–2014

Age group	Setting	Proportion of episodes (%)^a^	Mean cost per episode (US$) (95% CI)
Overall	All		218.3 (217.7–218.9)
	Hospitalized	0.1	1592.9 (1422.0–1763.8)
	ED/UC	10.7	329.6 (328.2–331.0)
	Outpatient	91.2	199.0 (198.4–199.6)
< 1 y	All		344.2 (341.0–347.4)
	Hospitalized	0.2	1977.9 (1289.3–2666.5)
	ED/UC	8.8	528.3 (520.6–536.0)
	Outpatient	95.1	309.8 (306.8–312.8)
1 y	All		338.2 (335.3–341.1)
	Hospitalized	0.1	1780.6 (1401.0–2160.2)
	ED/UC	10.1	504.5 (498.2–510.8)
	Outpatient	93.6	304.6 (301.7–307.5)
2–4 y	All		248.1 (246.4–249.8)
	Hospitalized	0.1	1480.5 (1205.2–1755.8)
	ED/UC	9.5	388.9 (385.1–392.7)
	Outpatient	92.8	226.1 (224.3–227.9)
5–17 y	All		186.1 (185.0–187.2)
	Hospitalized	0.1	1465.5 (1087.8–1843.2)
	ED/UC	10.4	293.6 (291.4–295.8)
	Outpatient	91.0	170.1 (169.0–171.2)
18–49 y	All		177.6 (176.6–178.6)
	Hospitalized	0.1	1737.1 (1312.5–2161.7)
	ED/UC	14.4	278.8 (276.7–280.9)
	Outpatient	87.3	156.5 (155.5–157.5)
50–64 y	All		188.8 (187.3–190.3)
	Hospitalized	0.1	1844.9 (1359.4–2330.4)
	ED/UC	8.5	274.5 (269.9–279.1)
	Outpatient	92.7	176.9 (175.4–178.4)
65–74 y	All		201.4 (196.3–206.5)
	Hospitalized	0.3	1257.3 (320.9–2193.7)
	ED/UC	5.7	269.8 (253.1–286.5)
	Outpatient	95.3	191.3 (187.1–195.5)
75–84 y	All		206.9 (200.4–213.4)
	Hospitalized	0.5	1011 (573.8–1448.2)
	ED/UC	4.7	318.2 (284.3–352.1)
	Outpatient	96.2	194.4 (188.4–200.4)
≥ 85 y	All		218.9 (208.3–229.5)
	Hospitalized	0.8	704.0 (350.9–1057.1)
	ED/UC	4.1	407.9 (340.3–475.5)
	Outpatient	96.5	203.8 (194.1–213.5)

*Abbreviations*: *CI* confidence interval, *ED* emergency department, *UC* urgent care

^a^Patients may have received several services, so total may be > 100%

## Discussion

This study showed that AOM-associated healthcare utilization and costs in the US remain substantial and changed little between 2008 and 2014. Children < 5 years of age had the highest rates of AOM. More than 90% of the AOM cases were seen in the outpatient setting and, other than rare hospitalizations, the rest were seen in ED/UC settings.

Randomized clinical trials have shown 0%–9% efficacy of PCV7 vs. all-cause AOM, suggesting that it would reduce AOM incidence, but database studies have not consistently indicated a decrease in AOM incidence since PCV7 was introduced in 2000 [6]. Between 2008 and 2014, incidence rates decreased slightly for children < 1 year of age but increased for adults, especially those older than 65 years. We also observed a small increase in the proportion of cases seen in ED and UC settings and slight change in the regional distribution of cases. Overall, however, the total incidence of AOM changed little and remained primarily an illness of young children. This suggests that PCV13, introduced in 2010, has not provided direct or indirect benefits beyond those already provided by PCV7.

Like *S. pneumoniae*, non-typable *H. influenzae* is a principal cause of AOM [3]. *H. influenzae* is normally a commensal microorganism present in the respiratory tract. Since *H. influenzae* type b vaccine was introduced in the late 1980s, non-typeable H. influenzae has become dominant [11]. Data collected between 2008 and 2010 in Rochester, New York, indicated that non-typable *H. influenzae* accounted for approximately the same proportion of AOM (28–34%) as *S. pneumoniae* (26–36%) [7]. The combined non-typeable *H. influenza*e protein D-pneumococcal polysaccharide vaccine is not available in the US, and the *H. influenzae* type b vaccines routinely used in the US do not prevent illness caused by non-typeable strains [12]. Protein-based non-typeable *H. influenzae* vaccines are being developed and are hoped to reduce AOM, but they may not be available for some years [11, 13]. Influenza is another vaccine-preventable illness that can cause AOM, although influenza viruses are minor contributors to the overall burden of AOM, so vaccination against influenza results in only a small reduction in AOM incidence [14].

AOM is one the illnesses for which antibiotics are most commonly prescribed [15, 16], and antibiotic prescription rates per consultation for AOM remain high in most countries (~ 80% of all consultations), especially in children < 2 years of age [17]. Other analyses of MarketScan data revealed that antibiotics were prescribed for 58% of AOM visits in 1997–2004 [16] and 52%–66% of ambulatory AOM visits in 2000–2011 [18]. As shown elsewhere [19], the current analysis showed that AOM episodes in the US peaks during the winter months, likely due to increased activity of respiratory pathogens. Thus, antibiotic prescriptions for AOM can help account for the increase in antibiotic prescriptions and antibiotic resistance of *S. pneumoniae* during winter months [20–22]. Reducing AOM may therefore not only reduce healthcare utilization and costs but also the spread of antibiotic resistance.

The estimated cost per AOM episode during 2013–2014 was similar to past studies. In 1997, Kaplan et al. reported that the average total cost of treating an episode of AOM was $115.8 in the US [23]. Similarly, a 2012 analysis of data from a large pharmacy network in the US found that, for members < 20 years of age, the unadjusted average cost per AOM episode ranged from $102 to $255 depending on whether the case was seen in a retail clinic or office visit [24]. These costs, however, do not capture indirect economic consequences of AOM such as out-of-pocket costs or lost productivity.

The results of this retrospective study are strengthened by the multiple years covered and the large size and representativeness of the MarketScan database [9], which includes nearly 40 million individuals and more than 2.5 million cases of AOM each year. In addition, the results reflect real-world outpatient and inpatient settings. We therefore expect that the estimates of AOM healthcare resources use and costs are reliable.

Our study has several limitations. Accuracy of the estimates could have been affected by measurement errors or misclassifications. For example, patients could have been misclassified because of misdiagnosis or miscoding. Miscoding could have occurred if a provider mistakenly submitted the wrong code, used a less descriptive ICD-9-CM billing code, misclassified a condition, up-coded to maximize reimbursement, or omitted a diagnosed condition on the billing forms. Also, data for some individuals could have been missing if they did not use their insurance for medical or pharmacy encounters. Another limitation is that we were unable to compare healthcare utilization by vaccination status because the information is not routinely collected by the MarketScan database. Further, regions were defined according to US Census categories, so we were unable to obtain for alternative regions or sub-region. Finally, the study was limited to individuals insured commercially or through the Medicare program. Care should be taken when attempting to generalize these results to other AOM patient populations (other private insurance, other public insurance, or no insurance) who may have different rates and types of resource use due to different coverage.

## Conclusions

AOM-associated healthcare utilization and costs in the US remain substantial and changed little between 2008 and 2014. This suggests that AOM caused by non-vaccine strains of *S. pneumoniae*, along with non-typeable *H. influenzae* and other infections continue to be a significant burden, especially for young children. This burden of AOM translates to frequent antibiotic use and probably also to bacterial resistance to antibiotics.

## Abbreviations

AOM: Acute otitis media
CI: Confidence interval
ED: Emergency department
ICD-9-CM: International Classification of Diseases, Ninth Revision, Clinical Modification
PCV13: 13-valent pneumococcal conjugate vaccine
PCV7: 7-valent pneumococcal conjugate vaccine
UC: Urgent care

## References

[1] Monasta L, Ronfani L, Marchetti F, Montico M, Vecchi Brumatti L, Bavcar A (2012). Burden of disease caused by otitis media: systematic review and global estimates. PLoS One.

[2] Vergison A, Dagan R, Arguedas A, Bonhoeffer J, Cohen R, Dhooge I (2010). Otitis media and its consequences: beyond the earache. Lancet Infect Dis.

[3] Leung AKC, Wong AHC (2017). Acute otitis Media in Children. Recent Patents Inflamm Allergy Drug Discov.

[4] Grabenstein JD, Klugman KP (2012). A century of pneumococcal vaccination research in humans. Clin Microbiol Infect.

[5] Cohen R, Varon E, Doit C, Schlemmer C, Romain O, Thollot F (2015). A 13-year survey of pneumococcal nasopharyngeal carriage in children with acute otitis media following PCV7 and PCV13 implementation. Vaccine.

[6] Taylor S, Marchisio P, Vergison A, Harriague J, Hausdorff WP, Haggard M (2012). Impact of pneumococcal conjugate vaccination on otitis media: a systematic review. Clin Infect Dis.

[7] Casey JR, Kaur R, Friedel VC, Pichichero ME (2013). Acute otitis media otopathogens during 2008 to 2010 in Rochester, New York. Pediatr Infect Dis J.

[8] Hansen L (2017). The Truven health MarketScan databases for life sciences researchers. In.

[9] Zhou F, Shefer A, Kong Y, Nuorti JP (2008). Trends in acute otitis media-related health care utilization by privately insured young children in the United States, 1997-2004. Pediatrics.

[10] Karhade AV, Larsen AMG, Cote DJ, Dubois HM, Smith TR. National Databases for Neurosurgical Outcomes Research: Options, Strengths, and Limitations. Neurosurgery. 2017;[Epub ahead of print]. 10.1093/neuros/nyx408 28950367

[11] Duell BL, Su YC, Riesbeck K (2016). Host-pathogen interactions of nontypeable Haemophilus influenzae: from commensal to pathogen. FEBS Lett.

[12] Khan MN, Ren D, Kaur R, Basha S, Zagursky R, Pichichero ME (2016). Developing a vaccine to prevent otitis media caused by nontypeable Haemophilus influenzae. Expert Rev Vaccines.

[13] Murphy TF (2015). Vaccines for Nontypeable Haemophilus influenzae: the future is now. Clin Vaccine Immunol.

[14] Norhayati MN, Ho JJ, Azman MY (2017). Influenza vaccines for preventing acute otitis media in infants and children. Cochrane Database Syst Rev.

[15] Hersh AL, Fleming-Dutra KE, Shapiro DJ, Hyun DY, Hicks LA (2016). Frequency of first-line antibiotic selection among US ambulatory care visits for otitis media, sinusitis, and pharyngitis. JAMA Intern Med.

[16] Fleming-Dutra KE, Hersh AL, Shapiro DJ, Bartoces M, Enns EA, File TM (2016). Prevalence of inappropriate antibiotic prescriptions among US ambulatory care visits, 2010-2011. JAMA.

[17] Haggard M (2011). Poor adherence to antibiotic prescribing guidelines in acute otitis media--obstacles, implications, and possible solutions. Eur J Pediatr.

[18] McGrath LJ, Becker-Dreps S, Pate V, Brookhart MA (2013). Trends in antibiotic treatment of acute otitis media and treatment failure in children, 2000-2011. PLoS One.

[19] Stockmann C, Ampofo K, Hersh AL, Carleton ST, Korgenski K, Sheng X (2013). Seasonality of acute otitis media and the role of respiratory viral activity in children. Pediatr Infect Dis J.

[20] Suda KJ, Hicks LA, Roberts RM, Hunkler RJ, Taylor TH (2014). Trends and seasonal variation in outpatient antibiotic prescription rates in the United States, 2006 to 2010. Antimicrob Agents Chemother.

[21] Sun L, Klein EY, Laxminarayan R (2012). Seasonality and temporal correlation between community antibiotic use and resistance in the United States. Clin Infect Dis.

[22] Dagan R, Barkai G, Givon-Lavi N, Sharf AZ, Vardy D, Cohen T (2008). Seasonality of antibiotic-resistant streptococcus pneumoniae that causes acute otitis media: a clue for an antibiotic-restriction policy?. J Infect Dis.

[23] Kaplan B, Wandstrat TL, Cunningham JR (1997). Overall cost in the treatment of otitis media. Pediatr Infect Dis J.

[24] Duncan I, Clark K, Wang S (2016). Cost and utilization of retail clinics vs. other providers for treatment of pediatric acute otitis media. Popul Health Manag.

